# Barriers to healthy nutrition: perceptions and experiences of Iranian women

**DOI:** 10.1186/1471-2458-12-1064

**Published:** 2012-12-10

**Authors:** Maryam Farahmand, Fahimeh Ramezani Tehrani, Parisa Amiri, Fereidoun Azizi

**Affiliations:** 1 Reproductive Endocrinology Research Center, Research Institute for Endocrine Sciences, Shahid Beheshti University of Medical Sciences, Tehran, Iran; 2 Obesity Research Center, Research Institute for Endocrine Sciences, Shahid Beheshti University of Medical Sciences, P.O. Box: 19395-4763, Tehran, Iran; 3 Endocrine Research Center, Research Institute for Endocrine Sciences, Shahid Beheshti University of Medical Sciences, Tehran, Iran

**Keywords:** Healthy nutrition, Barriers, Qualitative study, Non-communicable diseases

## Abstract

**Background:**

A sound understanding of community perceptions and experiences regarding barriers to a healthy diet is a prerequisite for the design of effective interventions aimed at prevention of diet-related non-communicable diseases (NCDs). This study focused on exploring barriers to healthy nutrition as experienced by women participating in the Tehran Lipid Glucose Study (TLGS).

**Methods:**

A grounded theory approach was used for analyzing the participants’ experiences and their perceptions regarding these barriers. Data collection was conducted through sixteen semi-structured focus group discussions, between 2008 and 2009. Participants were 102 women, aged 25-65 years, selected and recruited from the TGLS cohort. All interviews and focus group discussions were audio recorded and transcribed verbatim. Constant comparative analysis of the data was conducted manually according to the Strauss and Corbin analysis method.

**Results:**

The study revealed that the most important barriers to healthy nutrition were: 1) Interpersonal/cultural effects, 2) Lack of access to healthy foods, and 3) Food preferences.

**Conclusions:**

Understanding these barriers might contribute to existing literature by providing evidence from a different culture, and help design effective preventive strategies, and implement appropriate interventions among Tehranian families.

## Background

Following its rapid urbanization and socio-demographic changes, Iran is considered to be in the midst of a nutritional transition. Based on existing data, over the last two decades there has been a substantial shift in the food baskets of Iranian urban families. This shift is attributed to rising energy costs and a decrease in the quality of diet [1]. Studies have revealed that there is a consistent relationship between unhealthy diet and the emergence of a range of noncommunicable diseases (NCDs) including coronary heart disease, various cancers, diabetes mellitus, and various bone and joint diseases [2]. Today, these diseases have become the major cause of mortality and morbidity in Iran [3,4].

Research has clearly shown that lifestyle changes such as increased physical activity and healthy eating habits are the most affordable and cost-effective factors in controlling NCDs [5-7]. In response to these findings, the Tehran Lipid Glucose Study (TLGS) was designed to study the trend of NCDs and their risk factors, and to develop and implement strategies to reduce the risk of major NCDs and their consequences in the urban population of Tehran [3]. The results of primary nutritional assessment of TLGS participants showed that diets of most Tehranian adults lack the quality required and need improvement [8]. Preliminary evaluations of the TLGS nutritional interventions indicate only fat and carbohydrate intakes decreased after interventions, resulting in a reduction of plasma cholesterol and FBS levels. However, further results showed a significant increase in the body mass index among both the case and control groups [9]. Other findings also showed that the dietary patterns of most TLGS participants did not comply with the 2005 DGA and nutritional goals of WHO/Food and Agriculture Organization, especially for starchy vegetables, orange vegetables, lean meat, grains, and legumes [10]. These findings emphasized the necessity of more effective strategies to promote healthy eating habits among TLGS participants, initiating a significant move toward the reduction of the risk factors of the major NCDs [11].

With regard to healthy nutrition, previous studies suggest a sizable gap between what individuals have learned and what they practice [12-14]. Our previous data have consistently shown that dietary patterns may not accord with individuals’ knowledge of nutrition [15]. These findings reveal that attempts at improving diet by traditional strategies which have focused on public campaigns and health education have been slow. It is increasingly argued improving diets will require change in the broader personal and environmental factors that are at work in the creation of barriers to healthy nutrition and making the healthy choice difficult [16,17]. Understanding and addressing these factors is an essential part of any attempt to design and implement interventions directed at breaking down current barriers to healthy nutrition.

Women play a central role in shaping dietary patterns within their families [18], and as a prerequisite to the modification of the dietary patterns they follow, we must first understand their perceptions and experiences regarding the barriers to establishing healthy nutrition. Considering the limited reports available from Iran, this qualitative study was designed to explore barriers of healthy nutrition as perceived by women participating in TLGS. Understanding these barriers might contribute to existing literature by: 1) providing evidence from a different culture to explore the main underlying factors of unhealthy diet, and 2) help to design effective preventive strategies and implement appropriate interventions to improve nutritional pattern among Tehranian families.

## Methods

The current study was conducted within the framework of the TLGS, a large scale community based prospective study performed on a representative sample of residents of District-13 of Tehran, capital of Iran. Details of the rationale and design of the TLGS have been published elsewhere [3]. The TLGS has two major components: Phase 1 was a cross-sectional prevalence study of NCDs and their associated risk factors implemented from 1999 to 2001; Phase 2 is a prospective follow-up study in which NCD risk factors are measured approximately every 3 years. Following baseline collection of data, the intervention phase of the study was designed to improve healthy lifestyle and prevent NCD risk factors [11].

### Participants and data collection

This was a qualitative study conducted between January 2008 and February 2009. Participants were 122 women, aged 25-65 y, who were participated in the fourth prospective follow-up of the TLGS. They were a heterogeneous group from different occupational statues, education levels and marital statuses (Table 1). The criteria for selection of participants were participating in the TLGS and willingness to share their experiences. Participants were selected and recruited to the study using the TLGS data bank. The main researcher contacted the potential participants to explain the objectives and process of the current research. If the participants agreed to take part in the research, a focus group was scheduled. Overall, sixteen focus group discussions were conducted; with an average of 7 participants per group and a maximum of 12 (just one of the focus groups was conducted with 12 participants). To obtain participants views regarding their sociodemographic backgrounds, fourteen focus groups were conducted to collect data and two more were done to ensure data saturation. All of the focus groups were conducted by the main researcher and assisted by an assistant moderator, lasted between 1 to 3 hours, and were conducted in a private room using a semi-structured guide consisting of open-ended questions, enabling respondents to fully explain their personal opinions, perceptions, and experiences. The assistant moderator was asked to help with the setting up of the interview room rather than to take responsibility for it, deal with late-comers, take notes throughout the discussions and monitor recording equipment.

**Table 1:** The study participants’ characteristics

	**Number**	**Percentages**
**Total**	122	100
**Education**		
Primary	72	59.0
Secondary	29	23.8
Higher	19	15.6
**Marital Status**		
Single	11	9.0
Married	105	86.0
Divorced/widowed	6	5.0
**Occupation**		
Employee	13	10.7
House wife/Unemployed	96	78.7
Volunteer worker	12	9.80

To begin, each participant was asked to explain the following: Their individual perceptions of and experiences with healthy nutrition; the way that they choose and prepare their daily foods; the factors influencing their unhealthy food choices, and effective ways to help people choose healthy foods. During the focus group discussions, notes were taken about nonverbal signals and issues related to the research question which arose and were considered in the data analysis. All focus groups were conducted, audiotaped, transcribed and analyzed in Farsi. To establish credibility, a qualified bilingual individual, competent in the professional terminology of the nutritional qualitative research, validated the quality and conceptual equivalence of the translation [19].

### Data analysis

Data were analyzed manually and guided by constant comparative analysis [20]. In this study, data collection and analysis were done simultaneously according to the grounded theory approach. Open, axial, and selective coding was applied to the data. During open coding, each transcript was reviewed by at least two authors and the data reduced to codes. Differences in coding were resolved via discussions. Codes that were found to be conceptually similar in nature or related in meaning were grouped into subcategories. In axial coding the intent was to clarify how the emergent subcategories were related to preliminary categories. Analytical tools included asking questions and making comparisons, and were utilized to find the properties of each concept. Interviewing was stopped when data saturation occurred; data saturation occurred when no more codes identified through the last couple of focus groups and when the emerged categories were “coherent”.

#### Data trustworthiness

In this study, credibility and confirmability of the data were established by in-depth prolonged engagement with participants, and member checking. Prolonged engagement with participants in the research environment allowed the researcher to gain the participant’s trust and a deeper understanding of their situations. The first question concerning credibility arises when making a decision about the focus of the study, selection of context, participants and approaches to gathering data. Choosing participants with various experiences increases the possibility of shedding light on the research question from a variety of aspects [21]. In our study, interviewee various age, marital status, educational level, and job status contributed to a richer variation of the phenomena under study. To confirm dependability, four faculty members conducted a second review. Results were also checked with some of the women, who did not participate in the research and they confirmed the fitness of the results as well. All research details including procedures, actions and decisions were documented for audit purposes.

### Ethics

Ethics committee of the Research Institute for Endocrine Sciences, Shahid Beheshti, University of Medical Sciences approved the study. Participants provided written informed consent before the beginning of focus group discussions and explicit permission was sought for audio taping.

## Results

Results centered on three main themes that emerged from the data: (i) Interpersonal/cultural effects (ii) Lack of access to healthy foods, and (iii) Food preferences. Interpersonal/cultural effects encompassed two sub-themes: Unhealthy behavioral modeling, and inappropriate prioritizing. Lack of access to healthy foods included five sub-themes: Inadequate knowledge/information, high cost, time limitations to prepare healthy foods, poor hygiene and limited variety of healthy foods. Last, but not least, food preferences included: Personal taste and preferences of other family members.

### Theme 1: Interpersonal/cultural effects

#### Unhealthy behavioral modeling

Most women participating in this study pointed out that unhealthy behavioral modeling that influenced by family, peers and community which could directly affect family eating patterns is one of the most important factors in shaping unhealthy eating habits, especially among adolescents and young adults. *“One reason that kids prefer to eat sausage and salami in school is that they want to eat the same things their friends eat.”* Another influential factor was found within the school community. A popular opinion voiced by study participants was the school canteen is selling unhealthy foods to the kids. *“I am a mother. I want my kid to always be healthy in school. I want my kid to prefer the sandwich that I prepare for him, stuffed with cheese and walnuts, but he prefers the salami sandwich sold in school. Whenever we tell him not to eat such food, he responds with this answer; ‘If it is bad, then it should not be sold in school.’”*

Following extensive urbanization and a common access to media, deceptive advertisements also promote consumption of unhealthy food, thereby fostering unhealthy eating habits. Most consumers, such as the adolescents and young adults targeted by these advertisements, are manipulated into consuming unhealthy foods because they are deceived by these artful and misleading advertisements. They succumb to the tempting appearance of the food, whether or not it was nutritious. “*Advertisement and packing is really important especially for children and adolescents, who are easily motivated to buy unhealthy foods and snacks by advertisements.”* Another participated stated *“Chips and other snacks such as cheese puffs, and elaborately packed pizzas are available almost everywhere, in stores, schools, and university canteens, all aimed at attracting the attention of the younger generation, who obviously prefer to buy these types of food.”*

#### Inappropriate prioritization

In addition to unhealthy behavioral modeling, our study participants have also revealed inappropriate prioritization as an important barrier to healthy nutrition. An example is the inappropriate prioritization of the family budget, wherein limited resources are spent on items other than quality food. For example: *“A problem is the family’s tradition, especially the housewives’. I know of a family that prefers to spend less for food and more money for household furniture, indicating that food is given lower priority than material belongings.”*

Another example voiced by participants as a barrier to healthy nutrition is the decreasing value and importance of housekeeping, which means that the daily performance of household chores has become less important, especially among the younger generation of the community. Convenience has gained priority over traditional household chores, and cooking is no exception. *“My daughter lives in her own house and I can see that due to her laziness, a list of restaurants has been posted on her refrigerator. Every time she feels hungry, she just calls up any of these restaurants to order her food.”*

Additionally, study participants reported the inclination to use prepared and new foods reflects changing values and preferences, especially among the younger generation, toward fast and modern foods, and away from traditional foods. *“Yesterday for lunch I prepared vegetable stew but everyone ignored the food. My little boy said, ‘If only it was pizza.’ My mother-in-law prodded him to eat by saying that the food, aside from being fresh, was also good for him, but still he refused to eat.”*

### Theme 2: Lack of access to healthy foods

#### Inadequate knowledge/information

As pointed out by a large group of participants, women’s lack of knowledge was an important barrier to healthy nutrition, as demonstrated by their inability to distinguish healthy foods: *“I only use solid oil (saturated oil) in preparing my foods because it is safer than liquid oil (unsaturated oils), which we don’t know the exact ingredients of.”* Another participated stated, *“It is not only sausage and salami that are considered unhealthy foods. Since I am diabetic, the best vegetarian foods most probably will be considered unhealthy for my condition. I really have no idea what is good or bad for me.”*

A number of participants cited a lack of knowledge with regard to proper methods of food preparation, specifically those methods that preserve a food’s nutritional qualities. “*For cooking Ghormesabzi (a famous Iranian stew) we need to chop the vegetables to very small pieces, and then completely fry and cook them for at least 4-5 hours.”* Similarly, another participant referred to traditional ways of food preparation and cooking methods that they learned from their mothers as a main reason of their lack of knowledge regarding healthy food preparation. *“Our mothers used to cook some of traditional foods in such a manner that the cooking process would take hours, throughout the night till morning, and this was considered healthy. Today it is considered unhealthy to cook food more than two hours since all the vitamins in it will be destroyed.”*

#### High cost of healthy foods

*“Healthy foods are more expensive”*, was a common statement among study participants. The increasing cost of food clearly hinders access to healthy foods. *“To be able to buy cheaper milk (fat content 2.5%) for 250 tomans a packet, I have to stand in the queue. There is, however, another kind of milk with a fat content of 1.5%. I know it is much better, but a packet costs 800 tomans! I know that if I spend 800 tomans for this milk it will be healthy for me, but on my budget I cannot afford this milk.”*

In addition to the high cost of the food itself, study participants believed that the preparation cost of traditionally prepared foods made them prohibitively expensive, prompting food suppliers to meet the needs of the community by eschewing traditional methods of preparation for those that are less expensive and less nutritional. By all standards, this is considered by participants to be a major barrier to healthy nutrition within the family. *“This problem is universal. If chicken and eggs are produced in traditional ways, this method will not be able to meet the need of the increasing population. Populations have increased so much and this is the root cause of problems worldwide.”*

#### Time limitations to prepare healthy foods

For a variety of reasons, our study participants considered time limitations to be a significant barrier to healthy nutrition. “Working women” is one of the reasons cited by participants. These women lack the time required to prepare healthy meals, despite their awareness regarding healthy foods. *“In my opinion, more women are being employed and they have little or no time at all. For this reason they are more inclined to consume fast foods. When we cannot find vegetable stew in the restaurants, we have no other choice except pizza.” “Well, let’s assume that I am employed and my office hours are until 5:00 pm. When I reach home I have to prepare dinner, attend to the needs of my kids, then go to sleep. When do I get enough time? If I have access to a site where I can learn how to prepare a fast, healthy meal, my problem will be solved.”*

Furthermore, all women participating in this study (including housewives and those employed) unanimously stated that traditional foods are more healthy, but preparing and cooking them is more time consuming and difficult. Therefore, they believed that “the extra time needed to prepare a healthy meal” is a barrier to a healthy diet and increased the trend for families to consume fast foods. *“Homemade traditional foods are much better, but are very time consuming, and we cannot devote much of our time to cooking. However, ready-made foods can be prepared easily and quickly, leaving us with extra time for other chores.”*

#### Poor restaurant hygiene

Study participants have pointed out that “lack of observance of proper hygiene by restaurants and food catering services” is a fundamental issue and another reason for the lack of access to a healthy diet. *“TV frequently shows restaurants or food caterers and confectionaries not observing proper hygiene. I have no definite idea of what is really happening inside, but I have no choice but to go there and eat, although I suspect it is unhealthy.”*

#### Limited variety of healthy foods

Finally, some participants believed that “the limited variety of healthy foods available” restricts the possibility of choosing healthy foods, thus creating a barrier to healthy nutrition. *“We are restricted in terms of healthy foods while there are so many other healthy food stuffs we could prepare and consume.”*

### Theme 3: Food preferences

#### Personal taste

Participants pointed out that “the inclination to eat unhealthy foods because it suits one’s taste” is considered another barrier to healthy nutrition. Some considered taste and food preferences as the most important factor effective in the selection and consumption of foods, especially among children and adolescents. *“Despite all the advertisements on TV to drink churned sour milk (doogh) instead of soft drinks, I always prefer soft drinks. No matter how I tried to control this habit; still, I like them.”* Another participant stated, *“Children prefer sausage and salamis rather than the homemade foods and always find them delicious.”*

Participants believed that taste preferences, which develop throughout their life do not take food quality into account, dominating their eating habits regardless of health issues. *“Eating rice is harmful for me and my husband, since both of us have heart problems, but we have become too used to eating rice and if rice were eliminated from our diet we would feel that our stomachs were not full. At home, eating rice is and has always been a habit.”*

#### Preferences of other family members

Study participants believed “differences in family food preferences” is the biggest challenge a mother can face when attempting to provide her family with a healthy diet. To provide a healthy diet that would cater to all food preferences in the family can be a confusing and daunting barrier to healthy nutrition. *“Sometimes in my house we will fight over food. For example, my daughter and I love celery stew, but my son and my husband dislike it. Or, we all like pasta but my husband hates it.”*

## Discussion

The purpose of the current study was to explore barriers to healthy nutrition as experienced by women participating in the Tehran Lipid and Glucose Study (TLGS). The most prominent barriers faced by Iranian families were attributed to interpersonal and cultural factors which intensively affect their nutritional patterns. The women also reported problems with access to healthy foods and the food preferences of their family members, barriers, which reduced their motivation to modify their family’s unhealthy behaviors and hindered related interventions for NCD prevention from achieving successful results.

Consistent with previous findings [22], participants of this study believed that women’s inadequate knowledge regarding healthy foods and the healthful methods of preparing a variety of dishes are the two most important factors contributing to unhealthy nutrition among their families. With regard to the relationship between knowledge and awareness of eating habits, studies reveal different results. In one study, a strong and positive relationship was found between knowledge of nutrition and an individual’s functioning [1]. Other studies showed that students with more knowledge of the nutritional benefits of fruits and vegetables are more careful to include these foods in their diets [18,23]. However, several studies have shown that a person’s knowledge regarding nutrition is not necessarily the reason for their choice of healthy foods [23-25]. Increasing evidence documents the effects of physiologic needs, the person’s self-perception, food preferences, friends’ behaviors, and religious factors on an individual’s dietary behaviors [26].

According to our findings, the unhealthiest nutritional patterns in Iranian families are influenced by their children’s food preferences. Existing data show that there is an increasing tendency among Iranian children and adolescents toward Western dietary patterns that are largely defined by the consumption of high calorie snacks and fast foods [27]. As in many other countries, the attractive and colorful packaging of these foods, despite their poor nutritional values, is one factor inducing change in Iranian children’s food choices [28], a change reflected in an increasing trend toward fast foods and high calorie snacks.

Results of this study also showed low income as an important factor that could affect food choices among Iranian families. The relatively low cost of fast foods and high calorie snacks has made these foods more easily accessible to Iranian people [1]. Previous studies have similarly found cost to be one of the most important barriers to dietary adherence [29]. Existing data show that over the last two decades nutritional transition in Iran has been influenced by rising food prices [1]. However current findings imply that unhealthy diet is not entirely an income-driven issue; time limitations to the preparation of healthy food is noteworthy, particularly for working mothers, as is a lack of access to educational programs providing instructions for preparation healthy meal. Several studies report findings similar to ours [30-33].

In this study food preference, including personal taste and preferences of other family members was considered a barrier to healthy nutrition. Consistent with our findings, preferences of family members were among the most prominent barrier to implementing healthy dietary changes among immigrant Pakistani women [34].

This study was a qualitative study and the findings can provide a deep understanding of women’s perceptions of barriers to healthy nutrition among Iranian families, findings which could not be achieved through quantitative studies. Variety in sampling was an advantage of the current study. Participants of the study belonged to different socio-economical backgrounds and different occupation groups. Voluntary participation made room for exclusion of the experiences of those who did not wish to participate in the study for any reason. Also, all participants were selected from an urban community. Therefore our findings do not reflect barriers to healthy nutrition in rural communities. Complementary studies are recommended in these areas.

## Conclusions

Results from this study have indicated barriers to healthy nutrition as perceived by a group of Eastern-Mediterranean women, participants of the Tehran Lipid Glucose Study. Implementation of effective educational programs to increase public nutritional knowledge and appropriate policy-making seem prerequisite to establishing healthy eating patterns among Iranian families. Findings of the current study may help in the designing of healthy nutrition programs in the future by providing a realistic perspective of the current situation.

## Competing interests

The authors declare that they have no competing interests.

## Authors’ contributions

MF, PA, FRT and FA designed the study, collected and analyzed the data, and wrote the manuscript. All authors read and approved the final manuscript.

## Pre-publication history

The pre-publication history for this paper can be accessed here:

http://www.biomedcentral.com/1471-2458/12/1064/prepub
